# Tailored Metal‐Porphyrin Based Molecular Electrocatalysts for Enhanced Artificial Nitrogen Fixation to Green Ammonia

**DOI:** 10.1002/gch2.202300345

**Published:** 2024-06-03

**Authors:** Giorgia Salerno, Ottavia Bettucci, Norberto Manfredi, Luca Stendardo, Eleonora Veronese, Pierangelo Metrangolo, Alessandro Abbotto

**Affiliations:** ^1^ Department of Materials Science and Milano‐Bicocca Solar Energy Research Center (MIB‐SOLAR) University of Milano‐Bicocca Via Cozzi 55 Milano I‐20125 Italy; ^2^ Department of Information and Electrical Engineering and Applied Mathematics (DIEM) University of Salerno Invariante 12/B, Via Giovanni Paolo II, 132 Fisciano (SA) I‐84084 Italy; ^3^ Department of Chemistry, Materials, and Chemical Engineering “Giulio Natta” Politecnico di Milano Via L. Mancinelli Milano 20131 Italy

**Keywords:** artificial nitrogen fixation, hydrophilic chain, intermolecular interactions, metal‐porphyrins, molecular electrocatalyst

## Abstract

Electrochemical nitrogen reduction (E‐NRR) is one of the most promising approaches to generate green NH_3_. However, scarce ammonia yields and Faradaic efficiencies (*FE*) still limit their use on a large scale. Thus, efforts are focusing on different E‐NRR catalyst structures and formulations. Among present strategies, molecular electrocatalysts such as metal‐porphyrins emerge as an encouraging option due to their planar structures which favor the interaction involving the metal center, responsible for adsorption and activation of nitrogen. Nevertheless, the high hydrophobicity of porphyrins limits the aqueous electrolyte–catalyst interaction lowering yields. This work introduces a new class of metal‐porphyrin based catalysts, bearing hydrophilic tris(ethyleneglycol) monomethyl ether chains (metal = Cu(II) and CoII)). Experimental results show that the presence of hydrophilic chains significantly increases ammonia yields and *FE*, supporting the relevance of fruitful catalyst‐electrolyte interactions. This study also investigates the use of hydrophobic branched alkyl chains for comparison, resulting in similar performances with respect to the unsubstituted metal‐porphyrin, taken as a reference, further confirming that the appropriate design of electrocatalysts carrying peripheral hydrophilic substituents is able to improve device performances in the generation of green ammonia.

## Graphical abstract

Metal‐porphyrin‐based electrocatalysts bearing peripheral hydrophilic pendants for E‐NRR exhibit enhanced NH_3_ yields and *FE* by two to three times compared with the unsubstituted corresponding metal‐porphyrins. This work paves the way for new design strategies for NRR electrocatalysts representing an essential step toward the search for more sustainable alternatives for ammonia production.

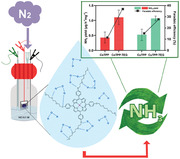

## 1 Introduction

Ammonia is undoubtedly one of the most used chemicals in modern civilization. The reason for ammonia's popularity lies in its widespread applications ranging from the production of nitric acid and fertilizers to explosives and synthetic fibers.^[^ ^1^ ^]^ Additionally, ammonia can be also exploited for the storage and transport of energy due to its high energy density of 3 kWh L^−1^.^[^ ^2^ ^]^ The Haber‐Bosch process, although developed in the early 20th century, is still the most significant process for producing ammonia on an industrial scale using nitrogen and hydrogen gases.^[^ ^3^ ^]^ Despite this process is considered one of the most impactful innovations of the 20th century, it is worth noting that it consumes a significant amount of energy (1 to 2% of the total anthropogenic energy in the world).^[^ ^4^ ^]^ Moreover, the hydrogen necessary for the process comes from steam‐methane reforming which requires the use of fossil fuels causing serious environmental implications.^[^ ^5^ ^]^ For all these reasons, efforts are being made to develop more sustainable and energy‐efficient methods for ammonia production such as the development of green hydrogen production implants.^[^ ^6^ ^]^ However, green hydrogen production requires a demanding 2‐step process, which involves water electrolysis powered by electricity from renewables which, in most countries, is still more expensive than methane.^[^ ^7^ ^]^ Hence, alternatives to directly convert atmospheric nitrogen into ammonia are required in order to avoid the use of hydrogen. To address this challenge nitrogen reduction reaction (NRR) via electrolysis (E‐NRR) and/or photoelectrochemically (Photo‐NRR) have been recently explored.^[^ ^8^ ^]^ Among the two approaches, photo‐NRR represents the most sustainable alternative to the energy‐intensive Haber‐Bosch process. However, drawbacks related to the low efficiencies, stability of the catalysts used, and poor reaction selectivity need to be overcome yet.^[^ ^9^ ^]^ E‐NRR in which the energy necessary for the reduction reaction is provided by the application of a potential difference is so far the most suitable direct way for sustainable production of ammonia.^[^ ^10^ ^]^ One of the challenges of this approach lies in the increase of electrocatalyst selectivity. Currently, NRR catalysts can be broadly classified into three categories: metal‐based materials,^[^ ^8^, ^11^ ^]^ metal‐free,^[^ ^11^ ^]^ and molecular catalysts.^[^ ^12^, ^13^ ^]^ Despite the numerous benefits of metal catalysts (i.e., high conductivity, good activation of the nitrogen, and the ability to bind to different reagents), some drawbacks related to the high costs, low natural abundance, and poisoning phenomena, limit their use.^[^ ^8^, ^10^, ^11^ ^]^ On the other side, the high versatility, mechanical flexibility, low cost, and excellent electrical conductivity of metal‐free electrocatalysts, make this class a promising alternative. However, their low number of catalytic sites is still a drawback to overcome.^[^ ^8^, ^13^, ^14^ ^]^ In this context, molecular materials, such as metal‐porphyrins and metal‐phthalocyanines, emerged as a very promising class of catalysts.^[^ ^15^ ^]^ Indeed, their insolubility in water electrolytes combined with the high stability in both acidic and basic conditions avoids the risk of dissolving them in the aqueous electrolytes.^[^ ^16^ ^]^ In addition to that, their planar structure favors reagents to coordinate axially with the metal centers, allowing effective interaction with the *d* orbitals of the metal which are responsible for adsorption and activation of nitrogen.^[^ ^17^ ^]^ Finally, a fine‐tuning of molecular structure of the catalyst allows a modulation of the electronic properties and consequently the increase in the NRR performances. However, a limit of this class of molecules lies in their high hydrophobicity which could limit the electrolyte‐catalyst interaction and consequently afford low NH_3_ yields. In other aqueous catalytic systems, the introduction of hydrophilic substituents catalyst structure to increase the electrolyte‐molecule interaction emerged as a winning strategy to overcome the above‐mentioned drawback.^[^ ^18^ ^]^ For this reason, in this work a new class of metal‐tetraphenyl porphyrins (M‐TPP), functionalized in the para position of the phenyl rings with hydrophilic tris(ethylene glycol) monomethyl ether (TEG) groups, (M‐TPP‐TEG) have been designed and synthesized. In fact, the TEG group has been successfully employed in many material science fields, including dye‐sensitized solar cells and nonlinear optics. More recently, the use of polyglycolic functionalities as substituents in organic molecules for dye‐sensitized hydrogen generation has been also reported.^[^ ^18^, ^19^ ^]^ The rationale behind such structural modification is to prevent the low wettability of the working electrode on which the catalyst is deposited as well as to promote a different molecular orientation of the metal‐porphyrins on the electrode substrate favoring the N_2_ fixation process. For a further confirmation of the linear relationship between the efficiency and electrolyte affinity, the same porphyrin scaffolds have been functionalized with branched hydrophobic alkyl chains, namely 2‐ethylhexyl (EH) units (M‐TPP‐EH). The effect of molecular staking and packing induced by the presence of different side chains has been also demonstrated and rationalized.

## 2 Results and Discussion

**Figure 1:**
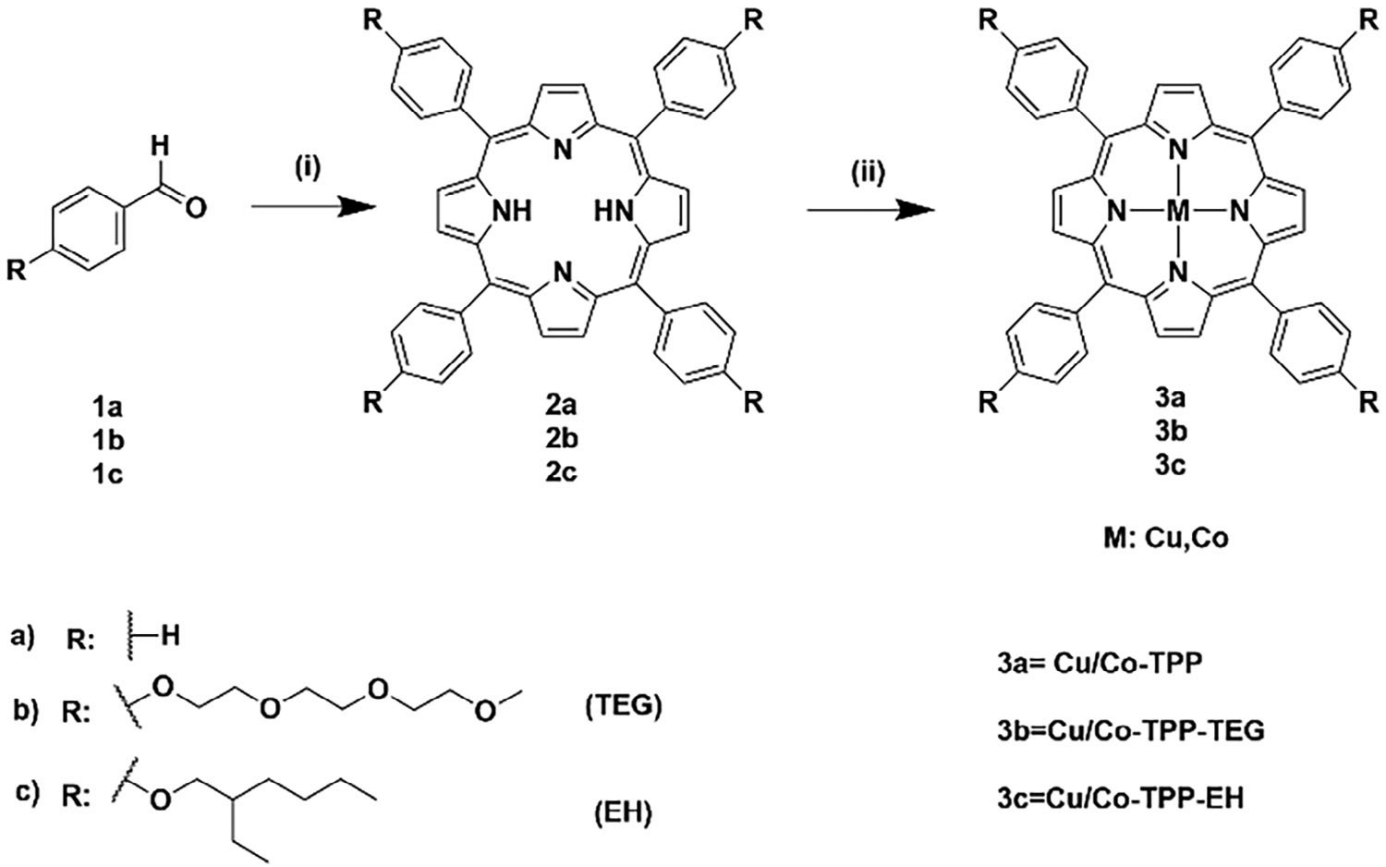
Cu/Co‐tetraphenylporphyrins synthetic pathway. (i) Pyrrole, propionic acid, reflux, 2.5 h; (ii) For M = Co: DMF, Co(CH_3_COO)_2_·4H_2_O, MeOH, 2 h, 110 °C; for M = Cu: DMF, Cu(CH_3_COO)_2_·H_2_O, MeOH, 2 h, 110 °C.

Novel functionalized metal‐tetraphenylporphyrins M‐TPP‐TEG (3b) and M‐TPP‐EH (3c), with M = Cu, Co, have been synthesized adapting a literature procedure.^[^ ^20^ ^]^ Their properties and electrocatalytic activity in artificial nitrogen fixation, compared to the corresponding reference compounds M‐TPP (3a), have been then evaluated. Starting from an appropriate amount of benzaldehyde (1a) functionalized with TEG (1b) or EH (1c) chains, the corresponding tetraphenylporphyrin TPP, TPP‐TEG, and TPP‐EH 2a‐c have been obtained. Then, 2a‐c have been submitted to metal coordination with a proper amount of Cu(II)/Co(II)‐acetate tetrahydrate to afford the desired products 3a‐c (**Figure** **1**). The novel metal‐porphyrins 3b and 3c have been chemically characterized through elemental analysis and UV–vis spectroscopy (Figures S1–S3 and Table S1, Supporting Information). Contact angle analysis was conducted to assess the hydrophilicity properties of the metal‐porphyrins film. The contact angles of deionized water drops on the surface are depicted in Figures S6 and S7 (Supporting Information). The hydrophobic Cu/Co‐TPP and Cu/Co‐TPP‐EH films exhibited contact angles higher than 110°, closely resembling the contact angle of the bare carbon paper (CP) surface, which measured 151° (vide infra). In contrast, the hydrophilic Cu‐TPP‐TEG and Co‐TPP‐TEG demonstrated contact angles of 29° and 39°, respectively. These results clearly indicate that the introduction of hydrophilic substituents significantly enhances the wettability of the photocatalyst surface. Detailed data are summarized in Figures S6 and S7, and Table S2 (Supporting Information). The electrochemical behavior has been evaluated recording cyclic voltammograms which showed similar redox behavior to that of the reference compounds 3a, previously reported in the literature.^[^ ^21^ ^]^ (Figures S4 and S5, Supporting Information).

**Figure 2:**
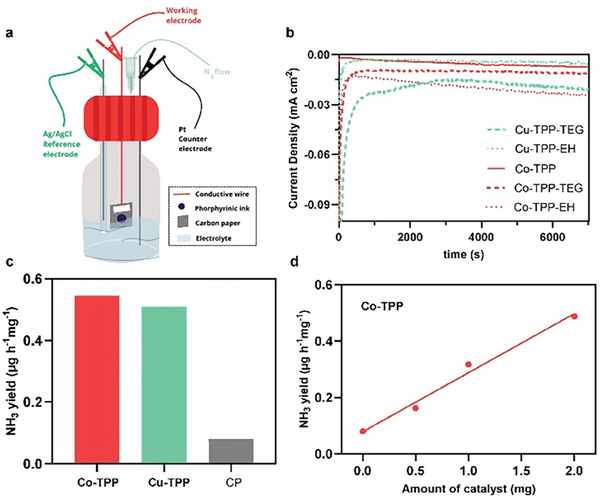
a) Experimental set‐up. b) Chronoamperometric curves of 3a–c. c) Average ammonia yield of the reference catalysts Cu‐TPP and Co‐TPP in comparison with pristine CP at −0.3 V versus RHE in a 0.1 m HCl electrolyte solution. d) Ammonia yield trend recorded upon increasing the amount of Co‐TPP electrocatalyst.

Electrochemical NRR experiments have been performed in 5 mL of a 0.1 m HCl electrolyte solution using a single neck electrochemical cell (**Figure** **2**a). The choice of an acidic electrolyte was justified to promote the hydrogenation process of nitrogen, thus enhancing the number of protons involved in the formation of NH_3_.^[^ ^15b^ ^]^ Reference and novel metal‐porphyrins 3a–c have been drop‐casted onto CP (loading: 1 mg cm^−2^), to obtain the corresponding working electrodes. An Ag/AgCl electrode and a Pt wire have been used as the reference and counter electrode, respectively.^[^ ^15b,c^ ^]^ The electrochemical cell has been kept under an inert atmosphere by continuously flowing high‐purity N_2_ gas (Nitrogen 5.0) during NRR investigation to avoid and eject any external contamination by air and other contaminants contained therein. Ion chromatography (IC) has been employed as a very accurate and high‐sensitivity method of choice to precisely quantify the amount of NH_3_ produced after 2 h of electrolysis. Compared with the spectroscopic methods commonly reported in nitrogen fixation studies, IC represents a direct detection method that provides several benefits such as low detection limit (down to 3 × 10^−7^ mol L^−1^), high reproducibility, and appropriate measurement precision.^[^ ^22^ ^]^ IC graphs of NRR experiments using 3a–c as electrocatalysts clearly showed a recognizable peak attributed to the NH_4_^+^ formed in the acidic environment following the production of NH_3_ (Figure S9, Supporting Information). Moreover, the chronoamperometric curves (recorded at −0.3 V vs RHE) exhibited constant current density over time, which indicates that all electrocatalysts presented the proper durability over the entire duration of the NRR runs (Figure 2b).^[^ ^15^, ^23^ ^]^ To further validate the experimental recordings and discard the undesired detection of NH_4_^+^ arising from other sources than electrocatalytic nitrogen fixation (e.g., from ammonia present as a contaminant in the air), a control experiment has been carried out using a working electrode made of pristine conductive CP in absence of electrocatalyst. Indeed, the control experiment showed that the NH_3_ yield produced by the pristine CP is significantly lower than that produced by the reference compounds 3a, thus proving that the major source of detected ammonia comes from the electrocatalytic NRR pathway via the metal‐porphyrin electrocatalysts (Figure 2c). Accordingly, the tiny amount of NH_3_ produced by the pristine CP that arises from contamination has been considered negligible. Regarding the catalyst, while the presence of nitrogen atoms in the structure could potentially lead to contamination, it is important to note that the number of nitrogen atoms is consistent across each structure. Therefore, if NH_3_ were to originate from the catalysts themselves, we would expect to observe similar NH_3_ production for each metal‐porphyrin tested. However, our findings reveal a discernible trend, indicating that the NH_3_ formed primarily originates from the N_2_ gas. To investigate in deeper details the key role of metal‐porphyrins in electrocatalytic NRR, the amount of catalyst on CP has been modified (loadings of 0.5 and 2 mg cm^−2^). Figure 2d evidences a trend in the NH_3_ yield which increases linearly with the concentration of the Co‐TPP (benchmark electrocatalyst). Such linear trend further confirmed that the amount of detected ammonia is strictly correlated to the amount of metal‐porphyrin catalyst.

**Figure 3:**
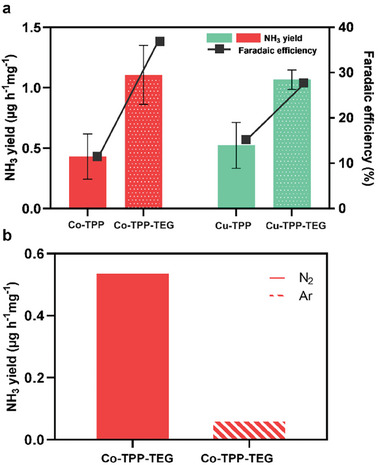
a) Amount of produced NH_3_ (left *y*‐axis) and FE (right *y*‐axis) using Cu/Co‐TPP 3a and Cu/Co‐TPP‐TEG 3b as electrocatalysts. b) Comparison of the amount of produced NH_3_ using Co‐TPP‐TEG 3b as electrocatalyst under N_2_ and Ar atmosphere.

Once validated the experimental set up and conditions, the electrocatalytic NH_3_ yields using of the novel metal‐porphyrin TEG derivatives 3b as electrocatalysts have been evaluated in comparison with the pristine (that is, without substituents on the phenyl rings of the porphyrin scaffold) metal‐porphyrin 3a. **Figure** **3** shows the average ammonia yield of the two TEG electrocatalysts 3b in comparison with the corresponding reference systems 3a. Indeed, the amount of produced ammonia with the investigated TPP‐TEG electrocatalysts, with both metal centers Co(II) and Cu(II), was significantly higher than that of the unsubstituted M‐TPP. In particular, the average ammonia yield for Co‐TPP‐TEG reached the value of 1.10 ± 0.07 µg h^−1^ mg^−1^_cat_, that is almost three times higher than that produced by Co‐TPP (0.43 ± 0.20 µg h^−1^ mg^−1^_cat_). Similarly, Cu‐TPP‐TEG reached an average ammonia yield of 1.06 ± 0,08 µg h^−1^ mg^−1^_cat_, thus doubling that produced by Cu‐TPP (0.52 ± 0.18 µg h^−1^ mg^−1^_cat_). These results show that Co‐TPP affords slightly better NH_3_ yields compared to Cu‐TPP, corroborating a previously published trend.^[^ ^15b^ ^]^ In both instances, the incorporation of hydrophilic TEG pendants substantially enhances NH_3_ yields, with comparable outcomes observed for both Co‐TPP‐TEG and Cu‐TPP‐TEG. Such favorable trend has been also confirmed by the *FE* calculated for 3b, which resulted to be 28 and 37% respectively, noticeably higher than those calculated for the corresponding references 3a (11% and 16%, respectively) (Figure 3 and Table S2, Supporting Information). The best‐performing metal‐porphyrin Co‐TPP‐TEG was also tested under an Ar atmosphere to further validate these results. The results (Figure 3b and Figure S11, Supporting Information) indicate a negligible level of ammonia production under Ar, thereby affirming that the NH_3_ generated predominantly originates from the fluxed N_2_.

**Figure 4:**
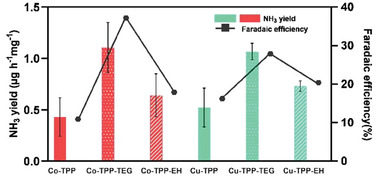
Overall comparison of produced NH_3_ (left y‐axis) and FE (right y‐axis) using unsubstituted Cu/Co‐TPP 3a, hydrophilic Cu/Co‐TPP‐TEG 3b, and hydrophobic Cu/Co‐TPP‐EH 3c as electrocatalysts.

The superior activity of the TEG electrocatalysts 3b supports the hypothesized critical issue related to the poor affinity of metal‐porphyrin based catalysts towards the aqueous medium. Indeed, the presence of proper hydrophilic chains in the para position of the peripheral phenyl rings of the porphyrin scaffold favors the electrolyte‐catalyst intermolecular interaction via hydrogen bonding, thus favoring the N_2_ fixation process to NH_3_ due to the formation of directional intermolecular interactions in aqueous media, as already demonstrated in photocatalytic H_2_ generation.^[^ ^18^ ^]^ At the same time, it is possible that the side chains might be endowed with higher degrees of freedom (free rotation along single bonds) suggesting a distinct arrangement of TEG‐derivatives 3b on CP compared to the reference metal‐TPP compounds 3a. To better understand the role of the hydrophilic chains, and more generally the effect of side chains, on the catalyst structure, the NRR performance of electrocatalysts 3c carrying conventional, hydrophobic, branched peripheral alkyl chain EH has been also investigated. The average ammonia yield for Co‐TPP‐EH and Cu‐TPP‐EH resulted to be 0.64 ± 0.20 and 0.73 ± 0.05 µg h^−1^ mg^−1^_cat_, respectively, that is considerably lower than those of the corresponding TEG derivatives 3b. Such behavior has been confirmed by *FE* which resulted to be 18 and 21% for Co‐TPP‐EH and Cu‐TPP‐EH, respectively (**Figure** **4**). These findings further confirmed the strategic role of peripheral hydrophilic chains in the molecular electrocatalysts in the promotion of efficient interfacial interaction between the different components of the electrocatalytic system and, eventually, in the enhancement of ammonia generation.

However, it is worth noting that both NH_3_ yields and FE using EH derivatives 3c are higher, though slightly, than those of the reference compounds 3a. Considering the error bars, we can conclude that the two performances are similar.

**Figure 5:**
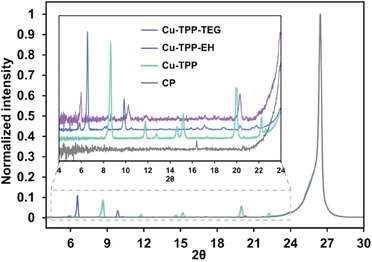
Powder X‐ray diffractograms (PXRD) of 3a–c Cu‐porphyrin thin films in comparison with bare carbon paper (CP).

The similar or slightly improved performances could be attributed to a favorable supramolecular arrangement of the catalyst molecules on the CP surface induced by the presence of terminal alkyl chains. We have previously demonstrated that a lower affinity of the branched alkyl units with the aqueous medium, which likely results in a coil‐like arrangement of the chains, can induce a distinct orientation of the catalyst units in H_2_ photocatalytic generation.^[^ ^18^ ^]^ To better understand this aspect, the thin film structures of 3a‐c were investigated using X‐ray diffraction (XRD) (**Figure** **5** and Figure S13, Supporting Information). Both Cu‐TPP and Co‐TPP produce highly crystalline films, with the XRD pattern exhibiting various diffraction peaks. Importantly, both films display the same structure of a single crystal of Cu‐TPP,^[^ ^24^ ^]^ as determined through a comparison with its simulated powder XRD pattern. It is noteworthy that the Cu‐TPP single crystal structure does not exhibit the typical planarity of the bare porphyrin core. Also, Cu/Co‐TPP‐TEG films are highly crystalline, although to a lesser extent than unsubstituted metal‐porphyrins 3a, showing an overall similar supramolecular organization.^[^ ^25^ ^]^ Similarly, Cu/Co‐TPP‐TEG films are crystalline, exhibiting the same long‐range order as those of 3a and 3b. However, in this case the aromatic stacking is lost, presumably due to the perturbation caused by the branched alkyl chains.

## 3 Conclusion

Two new classes of metal‐porphyrin based molecular electrocatalysts for NRR bearing different substituents in para to the peripheral phenyl rings (hydrophilic TEG derivatives **3b** and hydrophobic EH derivatives **3c**) have been synthesized and tested using a newly validated single‐neck experimental setup. The amount of produced ammonia and *FE* using TEG derivatives as electrocatalysts (Cu(II) and Co(II) complexes) resulted significantly enhanced (2‐3 times higher) compared to the corresponding reference metal‐porphyrins **3a** without substituents on the peripheral phenyl rings. Such a remarkable result supported our initial hypothesis based on the direct connection between the enhancement of the aqueous electrolyte‐hydrophilic catalyst intermolecular interaction and the nitrogen fixation process to green ammonia. The replacement of hydrophilic with conventional branched, hydrophobic, alkyl chain, resulting in lower performances (like those of unsubstituted metal‐porphyrins) further supports the strategic role of hydrophilic substituents in enhancing the electrocatalytic nitrogen fixation via proper electrolyte‐catalyst interactions. The findings have been also rationalized in terms of favorable supramolecular interaction of the catalyst molecules on the CP surface induced by the presence of the terminal alkyl chains. This work highlighted the key role and delicate balance on the presence and nature of substituents of the electrocatalyst main core in artificial nitrogen fixation to green ammonia, paving the way for new design strategies of NRR electrocatalysts and electrolyte solutions, including those based on nonaqueous green solvents such as deep eutectic solvents,^[^ ^18^, ^19^ ^]^ enabling even more variable and directional intermolecular interaction for cooperative enhancement.^[^ ^18^, ^26^ ^]^

## 4 Experimental Section

### General Information

Procedures for the synthesis of aldehydes and other intermediates are described in the Supporting Information. All the chemicals were commercially available and used without other purification. UV–vis spectra were recorded using a Jasco V‐570 spectrophotometer. NMR spectra were recorded with a Bruker Advance‐Neospectrometer operating at 400 MHz (1H). All the electrochemical measurements were carried out with a Bio‐logic SP‐240 and the detection of ammonia was carried out with an Ion Chromatograph Metrohm ECO‐IC with conductivity detector, mounted with cationic pre‐column Metrosep C6 S‐Guad /4.0 and cationic column Metrosep C6‐250/4.

### General Procedure for the Synthesis of Porphyrins **2a–2c**

Distilled pyrrole (1 equiv) was added into a reflux solution of propionic acid and the corresponding aldehyde (**1a‐c**) (1 equiv). The mixture was refluxed for 2.5 h and then cooled down to room temperature. The excess propionic acid was first distilled off under reduced pressure and the excess of solvent was then removed by rotary evaporation, resulting in a dark purple oil that was decanted with heptane. If necessary a purification by silica chromatographic column was carried out to afford the desired products as violet solids. Tetraphenylporphirin (**2a**) was synthesized according to the literature.^[^ ^20^, ^27^ ^]^

### 5,10,15,20‐Tetra[4‐[2‐[2‐(2‐methoxyethoxy]ethoxy]phenyl]porphyrin (**2b**)

Benzaldehyde **1b** (1.57 g, 5.85 mmol), pyrrole (0.40 mL, 0.39 g, 5.81 mmol), propionic acid (10 mL). After purification by chromatographic column (CHCl_3_/MeOH 9:1) product **2b** was obtained (263 mg, 0.21 mmol, 14%).^1^H‐NMR (CDCl_3_, ppm) *δ* = 8.85 (s, 8H), 8.10 (d, *J* = 8.1 Hz, 8H), 7.31 (d, *J* = 7.3 Hz, 8H) 4.44 (m, 8H), 4.07 (m, 8H), 3.90 (m, 8H), 3.76 (m, 8H), 3.63 (m, 8H), 3.61 (m, 8H), 3.43 (s,12H).

### 5,10,15,20‐(4‐(2‐Ethylhexyloxy)phenyl)porphyrin (**2c**)

Benzaldehyde **1c** (1.02 g, 4.35 mmol), pyrrole (0.30 mL, 0.29 g, 4.32 mmol), propionic acid 10 (mL). After the purification by chromatographic column (CH_2_Cl_2_/heptane 6:4) product **2c** was obtained (280 mg, 0.25 mmol, 23%). ^1^H NMR (CDCl_3_, ppm) *δ* = 8.87 (s, 8H), 8.11 (d, *J* = 8.6 Hz, 8H), 7.28 (d, *J* = 8.6 Hz, 9H), 1.95 – 1.90 (m, 5H), 1.62 (ddd, *J* = 17.9, 14.0, 6.5 Hz, 25H), 1.49–1.46 (m, 12H), 1.08 (s, 11H), 1.00 (s, 13H).

### General Procedure for the Synthesis of Metal‐Porphyrins **3a‐3c**

Reactions were carried out under a nitrogen flow to prevent oxidative phenomena. A solution of metal acetate (metal = Co(II) or Cu(II)) (1.8 equiv) in degassed MeOH (3 mL) was added to a solution of tetraphenyl porphyrin derivative **2a‐c** (1 equiv) in anhydrous DMF (10 mL). The resulting solution was stirred for 2 h at 110 °C. The mixture was treated with CH_2_Cl_2_ (30 mL) and the organic phase washed with H_2_O (4 × 20 mL) and dried over NaSO_4_. The excess of solvent was removed by rotary evaporation.

Co(II)‐tetraphenylporphyrin (Co‐TPP) (**3a**, M = Co)^[^ ^28^ ^]^ and Cu(II)‐tetraphenylporphyrin (Cu‐TPP) (**3a**, M = Cu)^[^ ^29^ ^]^ were prepared according to the literature.

### Co(II)‐TPP‐TEG (**3b**, M = Co)

Porphyrin **2b** (105 mg, 0.079 mmol), Co(CH_3_COO)_2_·4H_2_O (49 mg, 0.20 mmol). Product **3b** (M = Co): 68 mg, 0.051 mmol, 65%. Elemental analysis calcd. (%) for C_72_H_84_CoN_4_O_16_: C 65.49, H 6.41, N 4.24; found C 65.43; H 6.43; N 4.15.

### Cu (II)‐TPP‐TEG (**3b**, M = Cu)

Porphyrin **2b** (103 mg, 0.077 mmol), Cu(CH_3_COO)_2_·H_2_O (30 mg, 0.15 mmol). Product **3b** (M = Cu): 65 mg, 0.049 mmol, 64%. Elemental analysis calcd. (%) for C_72_H_84_CuN_4_O_16_: C 65.27, H 6.39, N 4.23; found C 65.24, H 6.85, N 4.45.

### Co(II)‐TPP‐EH (**3c**, M = Co)

Porphyrin **2c** (100 mg, 0.088 mol), Co(CH_3_COO)_2_·4H_2_O (44 mg, 0.18 mmol). Product **3c** (M = Co): 60 mg, 0.051 mmol, 58%). Elemental analysis calcd. (%) for C_76_H_92_CoN_4_O_4_: C 77.06, H 7.83, N 4.73; found C 77.21, H 8.04, N 4.90.

### Cu(II)‐TPP‐EH (**3c**, M = Cu)

Porphyrin **2c** (102 mg, 0.090 mol), Cu(CH_3_COO)_2_·H_2_O (23 mg, 0.12 mmol). Product **3c** (M = Cu): 50 mg, 0.042 mmol, 47%). Elemental analysis calcd. (%) for C_76_H_92_CuN_4_O_4_: C 76.76, H 7.80, N 4.71; found C 76.85, H 8.30, N 4.99.

### Preparation of **3a‐c** Ink

10 mg of electrocatalyst (**3a‐c**) and 40 µL of DuPont Nafion 5% solution were dispersed in 1 mL of water. This solution was then sonicated for 1 h to form a homogeneous catalyst ink.

### Preparation of Working Electrode

The conductive carbon paper (CP) substrate was cleaned and sonicated 5 times with ethanol and water. Then 100 µL of the porphyrin ink (loading of 1 mg cm^−2^) was drop‐casted onto the CP (area = 1 cm^2^).^[^ ^15b^ ^]^

### Powder X‐Ray Diffraction Analysis

The structural characteristics of the thin films of Cu‐TPP and Co‐TPP and their derivatives were investigated by powder XRD experiments that were carried out on a Bruker D8 Advance diffractometer operating in reflection mode with Ge‐monochromated Cu Kα1 radiation (*λ* = 1.5406 Å) and a linear position‐sensitive detector; with a 2*θ* range 5−40°, a step size 0.016° and exposure time 1.5 s per step.

### Electrochemical Measurements

All the electrochemical measurements were performed with a Bio‐logic SP‐240 room temperature using a 10 mL Ducan flask equipped with a perforable septum. The catalysts electrocatalytic NRR performance was evaluated in a standard 3‐electrode system and an electrolyte of 0.1 M HCl (5 mL) was used. CP with drop‐casted porphyrin ink has been used as the working, Ag/AgCl as the reference and a platinum wire as the counter electrode. In all measurements, the reference electrode was calibrated versus a reversible hydrogen electrode (RHE) with the following equation:^[^ ^15b^ ^]^

(1)

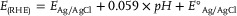

where *E*°_Ag/AgCl_ = standard reference with respect to NHE (for Ag/AgCl KCl_satd_ = 0.197 V a 25 °C)

Before starting chronoamperometry measurements, the system was degassed for 30 min with a constant flow of nitrogen. A constant nitrogen flow of 60 mL min^−1^ was maintained throughout the entire duration of the measurements.

### Detection of Ammonia

The concentration of produced NH_3_ was measured with an Ion Chromatograph. An aqueous solution of 4 × 10^−3^ m nitric acid and 0.7 × 10^−3^ m oxalic acid was used as the eluent for ammonium ion determination. The instrument returns a chromatogram in conductivity versus retention time. Whenever a cation is eluted at a given retention time, there is a change in conduction that leads to the generation of a peak whose area, after appropriate calibration, indicates the amount of ammonium ion in ppm. It is important to note that measurements for ammonia determination are very sensitive to accidental contamination. Therefore, it is necessary to analyze the electrolyte used for measurements before use and avoid external traces of ammonium in solution.

### Calculation of Ammonia Yield Rate and Faradaic Efficiencies (FE)

The rate of NH_3_ yield was calculated using the following equation:^[^ ^23^ ^]^

(2) $${\mathrm{NH}}_{3}\;\mathrm{yield}\;\;\left(\mu g/h\times {\mathrm{mg}}_{\mathrm{cat}}\right)=\frac{C\left({\mu g\;\mathrm{mL}}^{-1}\right)\times V\left(\mathrm{mL}\right)}{t\left(h\right)\times m\left({\mathrm{mg}}^{-1}\right)}$$

where *C* is the measured concentration, *V* is the volume of the electrolyte, *t* is the time and *m* is the mass of catalyst loading on CP.

*FE* of the NRR process is defined as the percentage of the amount of electric charge used for synthesizing NH_3_ over the total charge passed through the electrodes during the electrocatalytic procedure. Considering that each generated NH_3_ molecule requires 3 electrons, FE in 0.1 m HCl solution is calculated as:^[^ ^23^ ^]^

(3) $$\mathrm{FE}\;\left(\%\right)=\;\frac{3\times C\left({\mu g\;\mathrm{mL}}^{-1}\right)\times V\left(\mathrm{mL}\right)\times 10^{-6}\times F}{17\times Q\left(C\right)}$$

where 3 is the number of electrons required for each molecule of NH_3_, *F* is the Faradaic constant (96485.34 C mol^−1^), *V* is the volume of electrolyte, *C* is the measured concentration, and *Q* is the quantity of applied electricity.

## Conflict of Interest

The authors declare no conflict of interest.

## Supporting information

Supporting Information

## Data Availability

The data that support the findings of this study are available from the corresponding author upon reasonable request.
