# Supplementary material for: Tailored Metal‐Porphyrin Based Molecular Electrocatalysts for Enhanced Artificial Nitrogen Fixation to Green Ammonia
Source: Glob Chall. 2024 Jun 3;8(7):2300345. doi: 10.1002/gch2.202300345 (PMC11237181; doi:10.1002/gch2.202300345)
Supplement: Supplementary file 1 — Supporting Information [file GCH2-8-2300345-s001.pdf]

Global Challenges
Open Access

Supporting Information

for *Global Challenges.*, DOI 10.1002/gch2.202300345

Tailored Metal-Porphyrin Based Molecular Electrocatalysts for Enhanced Artificial Nitrogen Fixation to Green Ammonia

*Giorgia Salerno, Ottavia Bettucci, Norberto Manfredi, Luca Stendardo, Eleonora Veronese, Pierangelo Metrangolo and Alessandro Abbotto**

Supporting Information

Tailored Metal-Porphyrin Based Molecular Electrocatalysts for Enhanced Artificial Nitrogen Fixation to Green Ammonia

Giorgia Salerno, Ottavia Bettucci, Norberto Manfredi, Luca Stendardo, Eleonora Veronese, Pierangelo Metrangolo, and Alessandro Abbotto

Contents

Synthesis data for **3a (M=Co)**, **Co-TPP**..2

Synthesis data for **3a (M=Cu)**, **Cu-TPP**..3

Figure S1. UV-Vis absorption spectra of **TPP**, **Co-TPP** and **Cu-TPP** in $CHCl_3$ 3

Figure S2. UV-Vis absorption spectra of **TPP-TEG**, **Co-TPP-TEG** and **Cu-TPP-TEG** in $CHCl_3$ 4

Figure S3. UV-Vis absorption spectra of **TPP-EH**, **Co-TPP-EH** and **CU-TPP-EH** in $CHCl_3$ 4

Table S1. Absorption peaks of compounds **2a-c** and **3a-c** 5

Figure S4. CV **Co-TPP-TEG** (left) and **Cu-TPP-TEG** catalysts in a 0.1 M tetrabutylammonium perchlorate solution in dichloromethane as a supporting electrolyte using a glassy carbon working electrode, a Pt wire as a counter electrode $Ag/AgNO_3$ in 0.1 M tetrabutylammonium perchlorate solution in acetonitrile as pseudo-reference electrode. 5

Figure S5. CV **Co-TPP-EH** (left) and **Cu-TPP-EH** catalysts in a 0.1 M tetrabutylammonium perchlorate solution in dichloromethane as a supporting electrolyte using a glassy carbon working electrode, a Pt wire as a counter electrode $Ag/AgNO_3$ in 0.1 M tetrabutylammonium perchlorate solution in acetonitrile as pseudo-reference electrode. 6

Figure S6. Cross-sectional images of porphyrins film on carbon paper: a i-ii) **Cu/Co-TPP** b i- ii) **Cu/Co-TPP-EH** c i-ii) **Co/Cu-TPP-TEG** 6

Figure S7. Cross-sectional images bare carbon paper (**CP**). 7

Table S2. Calculated contact angle values. 7

Figure S8. Calibration curve IC. 7

Table S3. Value obtained from IC curves $NH4^+$ standard solution at different concentration 8

Figure S9. Ammonium-related peaks observed in IC chromatograms after 2 h. 8

Figure S10. Electrolyte IC chromatogram 9

Figure S11. Observed peaks in IC chromatograms after 2 h under Ar atmosphere. 9

Table S4. Ammonia yields and *FE* using **3a-c** as electrocatalysts. 9

Table S5. TON (after 2 h) and TOF values using **3a-c** as electrocatalysts. 10

Table S6. Comparison of properties with other porphyrins based electrocatalysts for E-NRR present in literature. 10

Figure S12. CV (multiple cycles) of the investigated porphyrins on CP electrode as a working electrode, a Pt wire as a counter electrode and Ag/AgCl 3.5 M KCl pseudo-reference electrode in 0.1 M HCl_{aq} 11

Figure S13. Powder X-Ray Diffractograms (PXRD) of **3a** and **3b** Co-porphyrin thin films in comparison with bare carbon paper (CP). 12

Figure S14. 1H-NMR spectrum of **TPP-TEG** in $CDCl_3$ (aromatic region). 13

Figure S15. ^{1}H-NMR spectrum of **TPP-TEG** in $CDCl_3$ (aliphatic region). 13

Synthesis data for 3a (M=Co), Co-TPP.

2a (117 mg, 0.19 mmol), $Co(CH_3COO)_2{\cdot}4H_2O$ (85 mg, 0.34 mmol). Product 3a (M = Co): 113 mg, 0.17 mmol, 88%). Elemental analysis calcd. (%) for $C_{44}H_{28}CoN_4$: C 78.86, H 4.20, N 8.34; found C 78.86, H 4.93, N 8.36.

Synthesis data for 3a (M=Cu), Cu-TPP.

2a (271 mg, 0.44 mmol), $Cu(CH_3COO)_2{\cdot}H_2O$ (144 mg; 0.72 mmol). Product 3a (M = Cu): 248 mg, 0,36 mmol, 81%. Elemental analysis calcd. (%) for $C_{44}H_{28}CuN_4$: C 77,91, H 4,46, N 8,26; found C 78.29, H 4,93, N 8.36. UV-Vis (THF): λ_{max} 415, 541 nm.

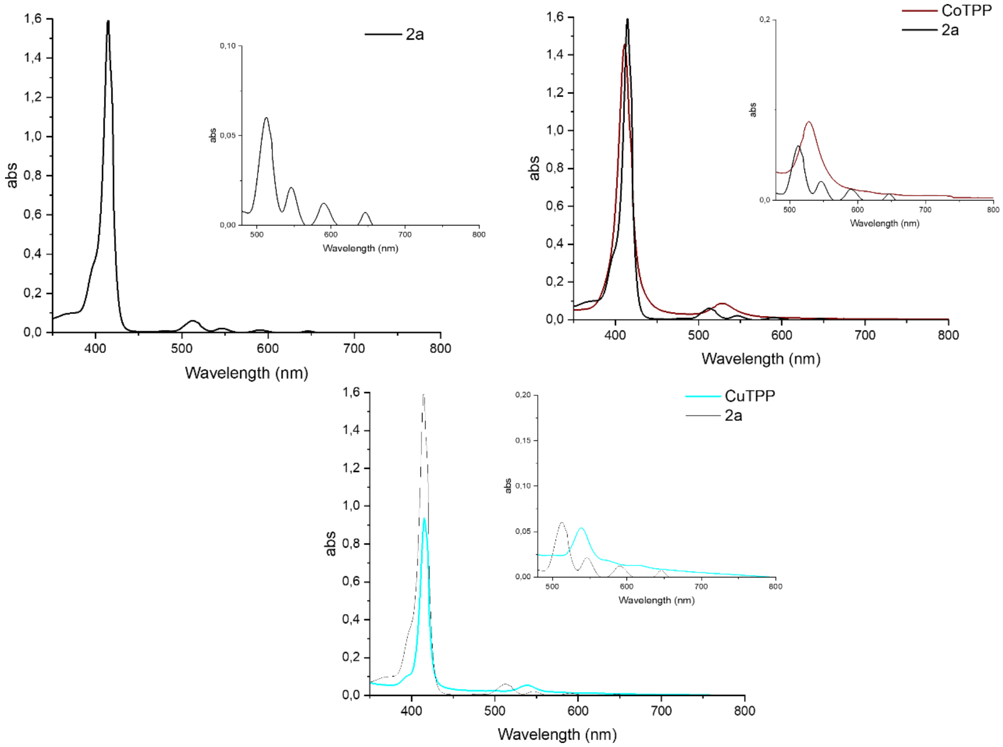

Figure S1. UV-Vis absorption spectra of TPP, Co-TPP and Cu-TPP in $CHCl_3$.

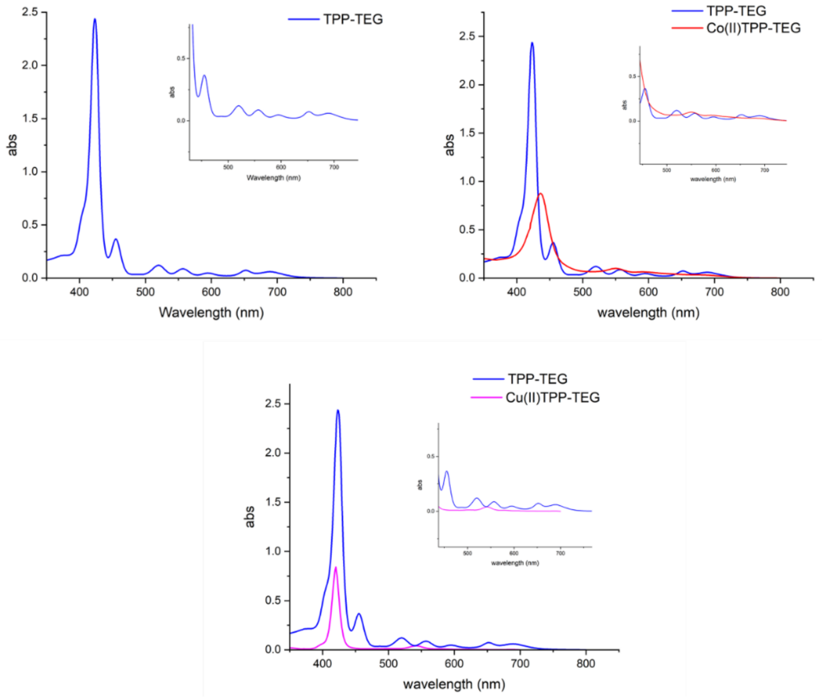

Figure S2. UV-Vis absorption spectra of TPP-TEG, Co-TPP-TEG and Cu-TPP-TEG in $CHCl_3$.

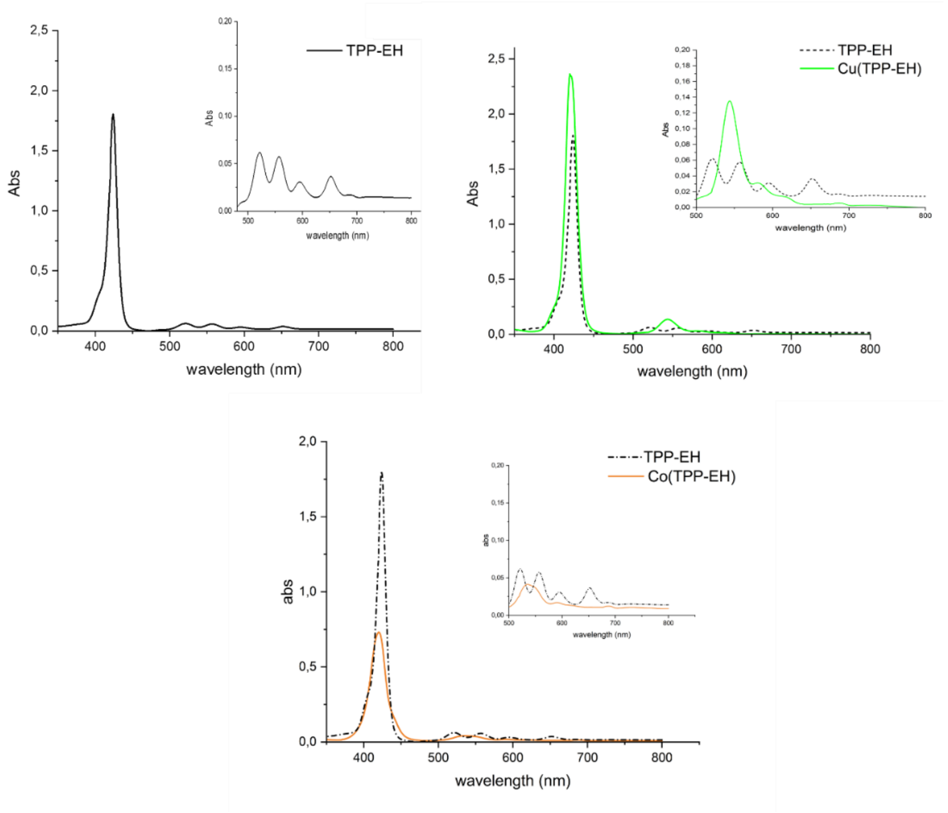

Figure S3. UV-Vis absorption spectra of TPP-EH, Co-TPP-EH and CU-TPP-EH in $CHCl_3$.

Table S1. Absorption peaks of compounds 2a-c and 3a-c.

Compounds	λmax (nm)	Solvent
**2a**	420, 517, 551, 592, 648	EtOH
**2b**	423, 455, 520, 556, 597, 652, 690	$CHCl_3$
**2c**	423, 521, 556, 595, 651	$CHCl_3$
**3a (M = Co)**	411, 528	CH_2Cl_2
**3a (M = Cu)**	415, 541	THF
**3b (M = Co)**	436, 550, 594	$CHCl_3$
**3b (M = Cu)**	420, 542	$CHCl_3$
**3c (M = Co)**	419, 535, 601	$CHCl_3$
**3c (M = Cu)**	419, 544, 587	$CHCl_3$

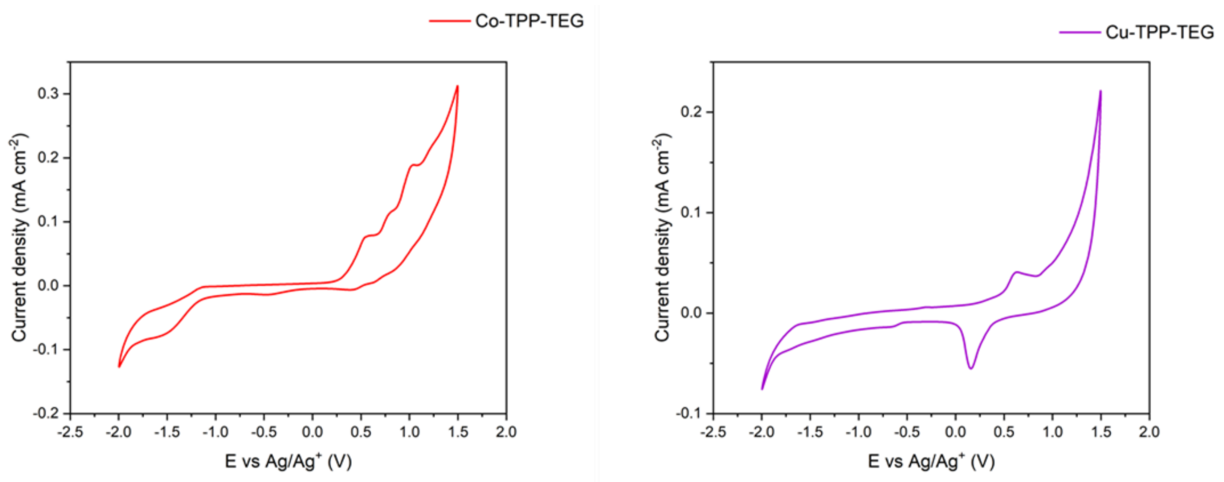

Figure S4. CV Co-TPP-TEG (left) and Cu-TPP-TEG catalysts in a 0.1 M tetrabutylammonium perchlorate solution in dichloromethane as a supporting electrolyte using a glassy carbon working electrode, a Pt wire as a counter electrode Ag/$AgNO_3$ in 0.1 M tetrabutylammonium perchlorate solution in acetonitrile as pseudo-reference electrode.

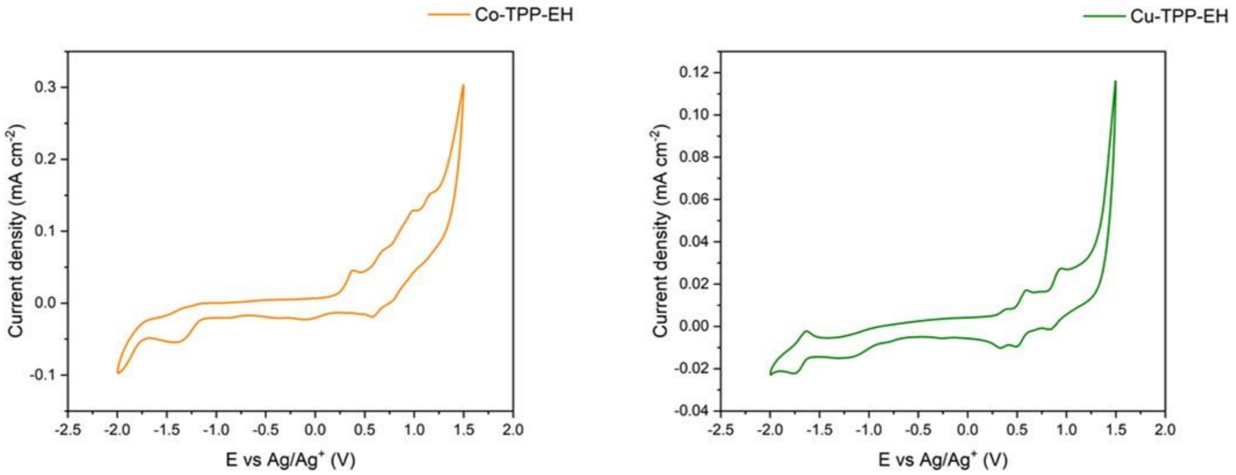

Figure S5. CV Co-TPP-EH (left) and Cu-TPP-EH catalysts in a 0.1 M tetrabutylammonium perchlorate solution in dichloromethane as a supporting electrolyte using a glassy carbon working electrode, a Pt wire as a counter electrode $Ag/AgNO_3$ in 0.1 M tetrabutylammonium perchlorate solution in acetonitrile as pseudo-reference electrode.

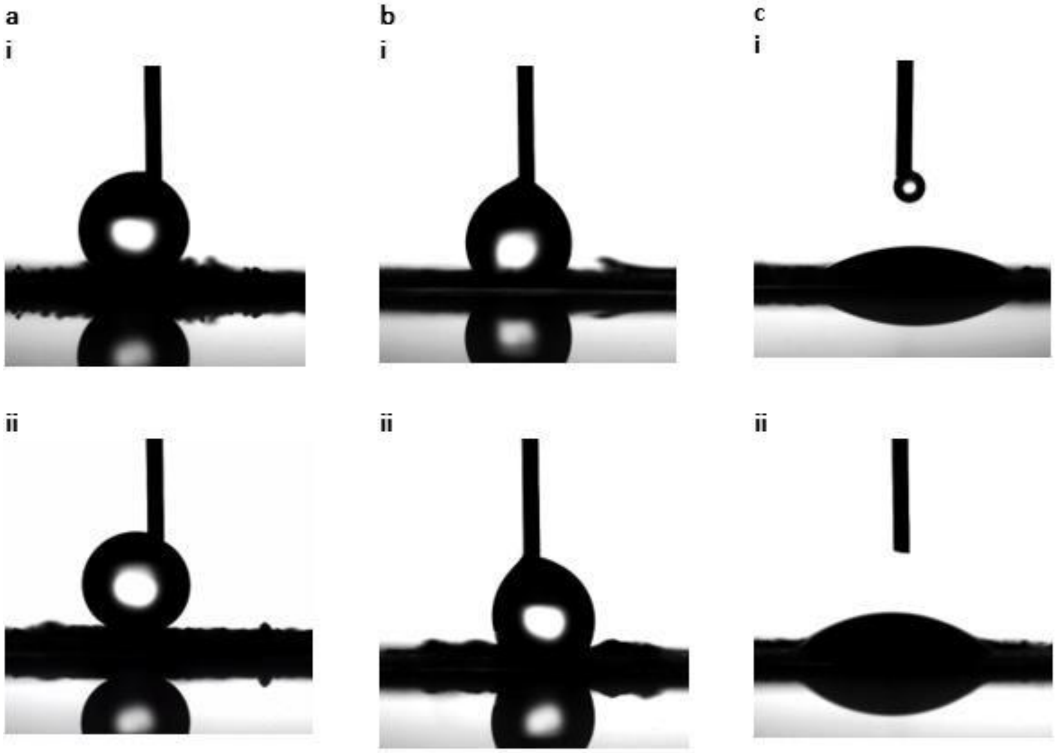

Figure S6. Cross-sectional images of porphyrins film on carbon paper: a i-ii) Cu/Co-TPP b i- ii) Cu/Co-TPP-EH c i-ii) Co/Cu-TPP-TEG.

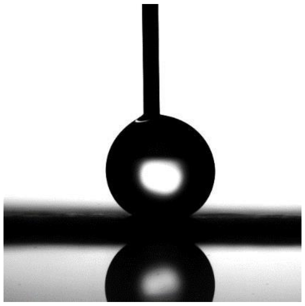

Figure S7. Cross-sectional images bare carbon paper (CP).

Table S2. Calculated contact angle values.

Electrocatalyst	Contact angle ϑc (°)
CP	151.35
**3a (M = Co)**	139.75
**3b (M = Co)**	29.7
**3c (M = Co)**	115.75
**3a (M = Cu)**	150.15
**3b (M = Cu)**	39.45
**3c (M = Cu)**	115.95

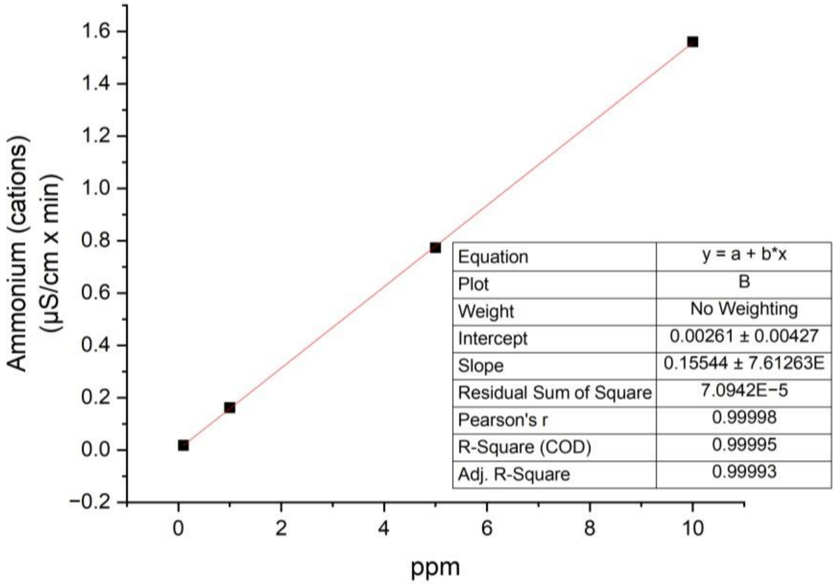

Figure S8. Calibration curve IC.

Table S3. Value obtained from IC curves $NH4^{+}$ standard solution at different concentration.

Sample type	Volume	Conc. (ppm)	Area
**NH_4^+ 10 ppm**	10.0	10	1.560
**NH_4^+ 5 ppm**	10.0	5	0.773
**NH_4^+ 1 ppm**	10.0	1	0.162
**NH_4^+ 0.1 ppm**	10.0	0.1	0.018

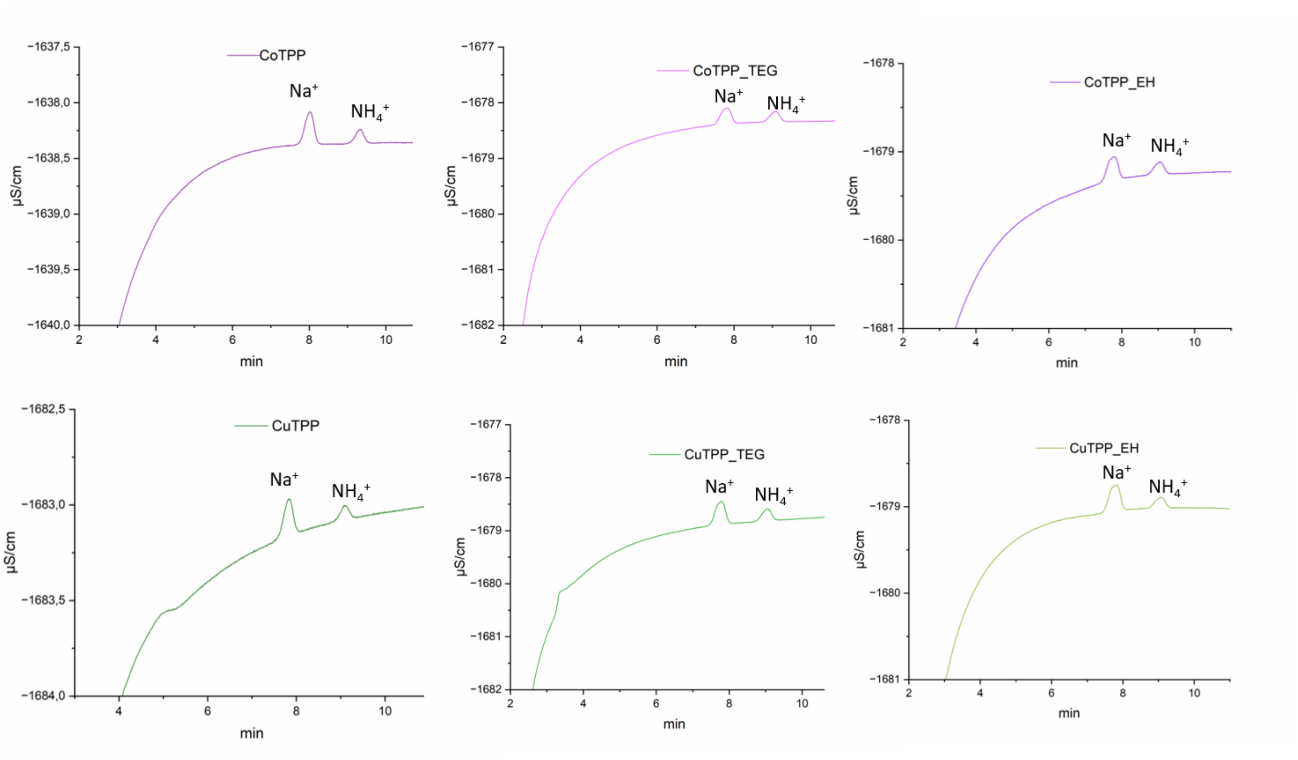

Figure S9. Ammonium-related peaks observed in IC chromatograms after 2 h.

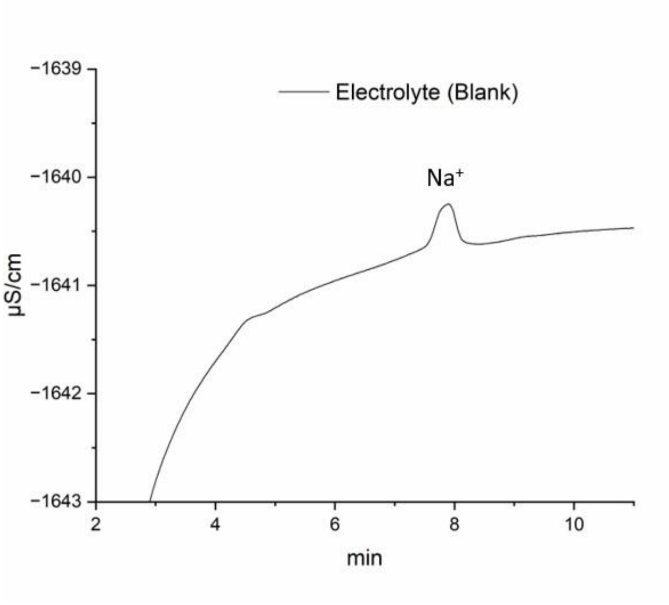

Figure S10. Electrolyte IC chromatogram.

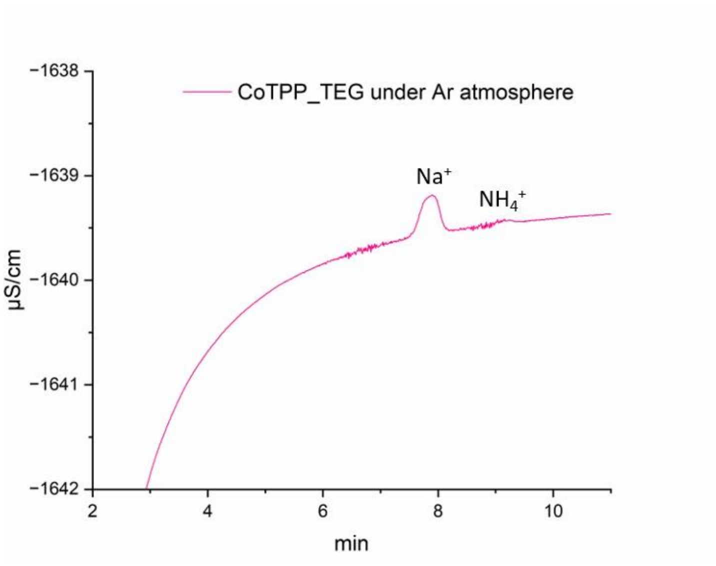

Figure S11. Observed peaks in IC chromatograms after 2 h under Ar atmosphere.

Table S4. Ammonia yields and *FE* using 3a-c as electrocatalysts.

Electrocatalyst	NH_3 yield (μg $h^{-1}mg^{-1}$)	FE(%)
**3a (M = Co)**	0.430 ± 0.19	10.9
**3b (M = Co)**	1.106 ± 0.07	16.2
**3c (M = Co)**	1.066 ± 0.08	37.3
**3a (M = Cu)**	0.523 ± 0.27	27.8
**3b (M = Cu)**	0.642 ± 0.21	17.9
**3c (M = Cu)**	0.731 ± 0.05	20.4

Table S5. TON (after 2 h) and TOF values using 3a-c as electrocatalysts.

Electrocatalyst	quantity of electrocatalyst (mg)	TON	TOF (sec^{-1})
**3a (M = Co)**	1	0.02	2.8 x 10^{-6}
**3b (M = Co)**	1	0.85	1.2 x 10^{-4}
**3c (M = Co)**	1	0.18	2.5 X 10^{-5}
**3a (M = Cu)**	1	0.05	6.7 x 10^{-6}
**3b (M = Cu)**	1	0.12	1.6 X 10^{-5}
**3c (M = Cu)**	1	0.25	2.5 X 10^{-5}

Table S6. Comparison of properties with other porphyrins based electrocatalysts for E-NRR present in literature.

Catalyst	NH_3 yield rate	FE	Ref.
FeTPPCl	18.3 μg h^{-1} mg_{cat}^{-1}	16.8%	[15c]
CoTPP	15.2 μg h–^{1} mg_{cat}^{-1}	11.4%	[15b]
MnPc/C	127.7 μgh^{-1} mg_{cat}^{-1}	35.3%	[a]
CoTPP-TEG	1.1 μg h^{-1} mg_{cat}^{-1}	37%	[this work]
CuTPP-TEG	1.1 μg h^{-1} mg_{cat}^{-1}	28%	[this work]

[a] Adalder, A.; Waghela, S. R.; Shelukar, S. A.; Mukherjee, N.; Das, S.; Ghorai, U. K., *Eng. Rep.* **2024,** *6* (1), e12705.

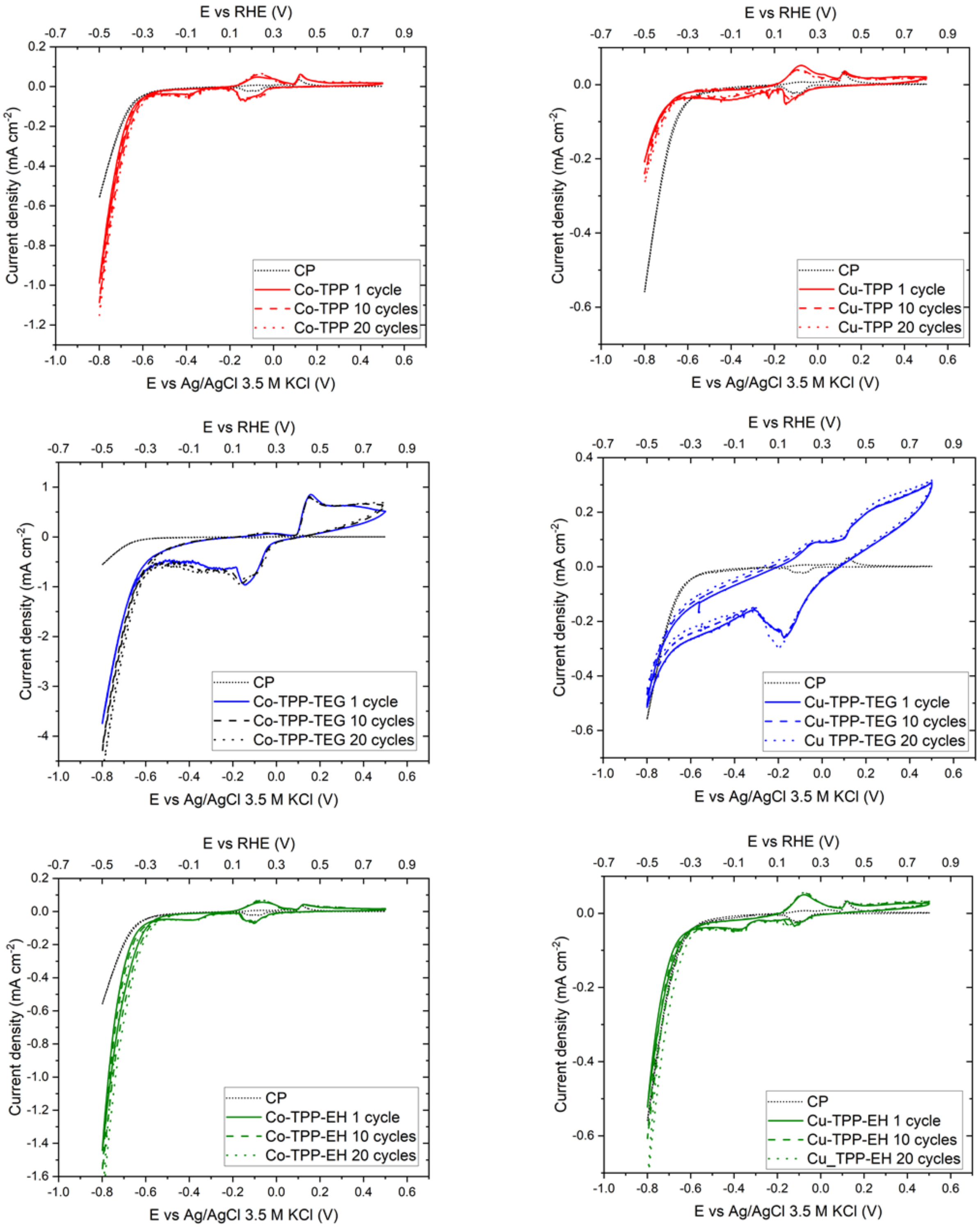

Figure S12. CV (multiple cycles) of the investigated porphyrins on CP electrode as a working electrode, a Pt wire as a counter electrode and Ag/AgCl 3.5 M KCl pseudo-reference electrode in 0.1 M HCl_{aq}.

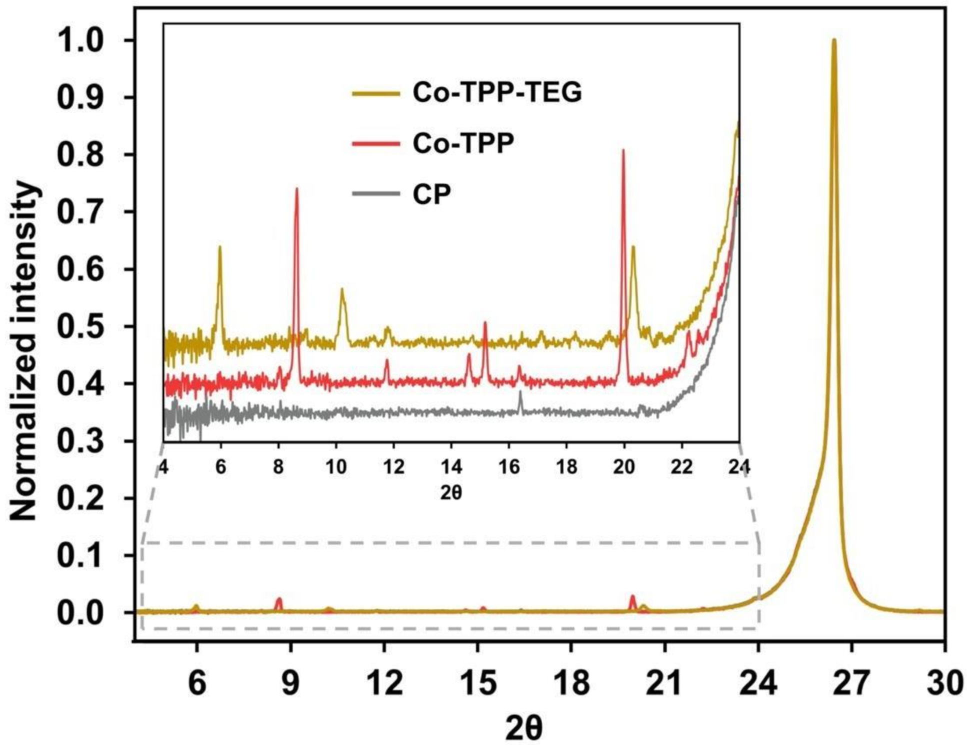

Figure S13. Powder X-Ray Diffractograms (PXRD) of 3a and 3b Co-porphyrin thin films in comparison with bare carbon paper (CP).

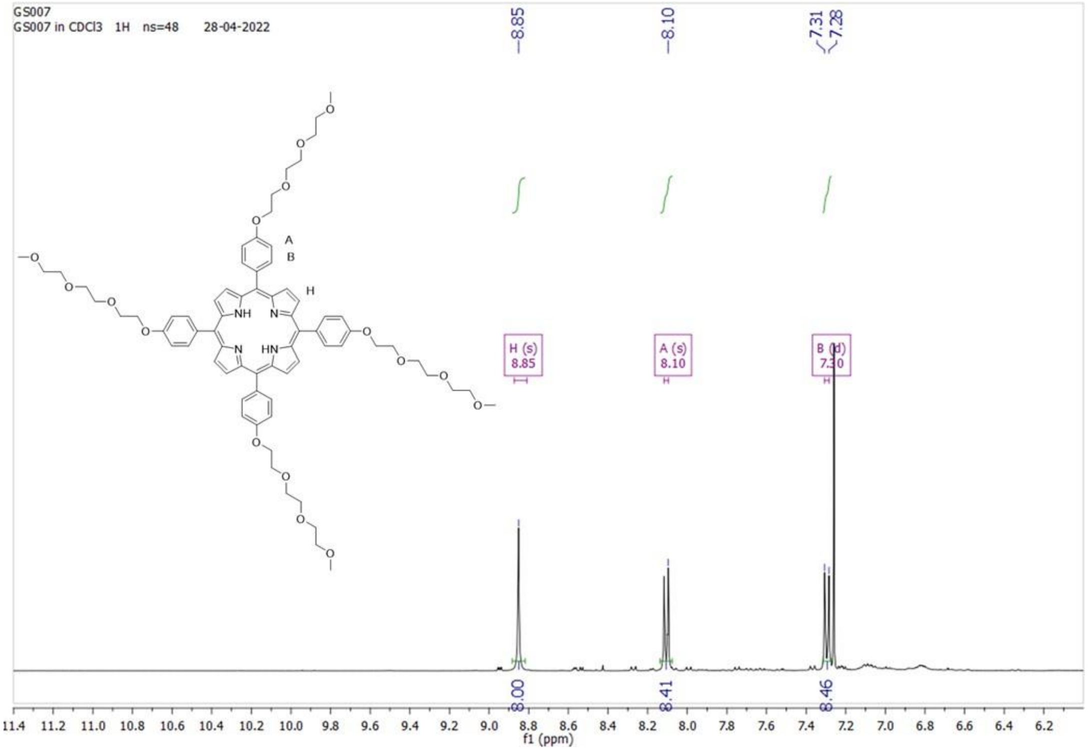

Figure S14. 1H-NMR spectrum of TPP-TEG in $CDCl_3$ (aromatic region).

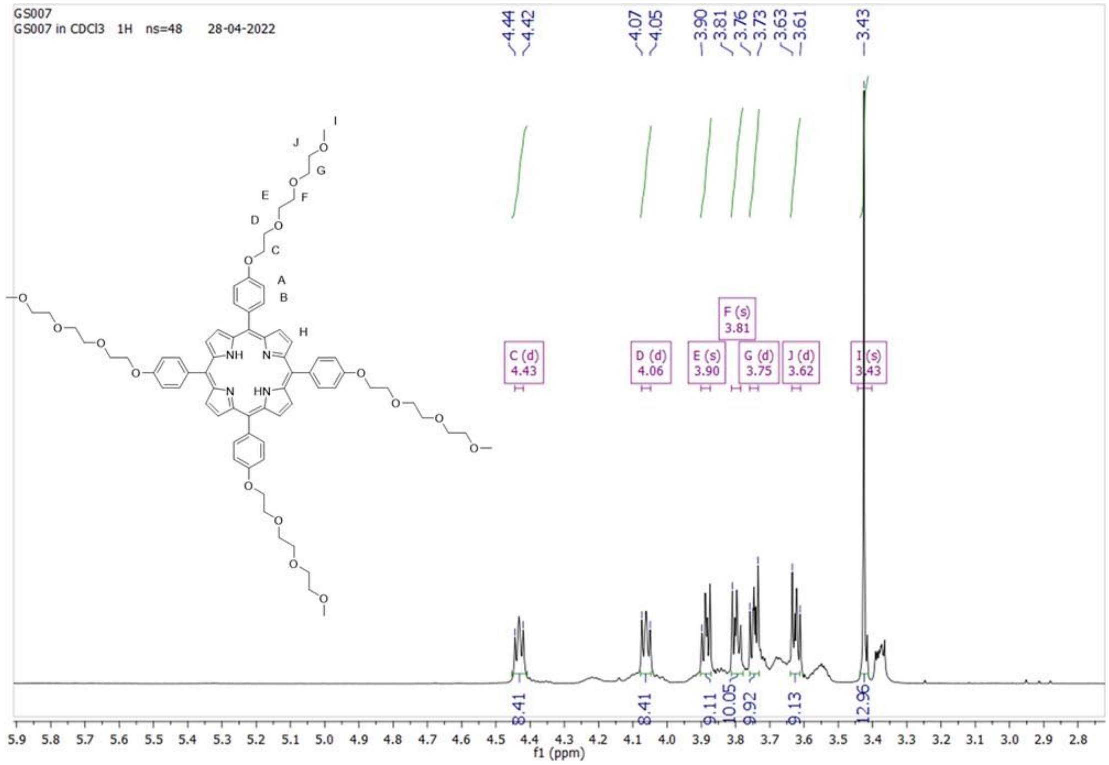

Figure S15. ^{1}H-NMR spectrum of TPP-TEG in $CDCl_3$ (aliphatic region).
